# Microneedle-Based Natural Polysaccharide for Drug Delivery Systems (DDS): Progress and Challenges

**DOI:** 10.3390/ph15020190

**Published:** 2022-02-03

**Authors:** Fouad Damiri, Nagavendra Kommineni, Samuel Ogbeide Ebhodaghe, Raviteja Bulusu, Vaskuri G. S. Sainaga Jyothi, Amany A. Sayed, Aeshah A. Awaji, Mousa O. Germoush, Hamdan S. Al-malky, Mohammed Z. Nasrullah, Md. Habibur Rahman, Mohamed M. Abdel-Daim, Mohammed Berrada

**Affiliations:** 1 Laboratory of Biomolecules and Organic Synthesis (BIOSYNTHO), Department of Chemistry, Faculty of Sciences Ben M’Sick, University Hassan II of Casablanca, Casablanca 20000, Morocco; berrada_moh@hotmail.com; 2 Center for Biomedical Research, Population Council, New York, NY 10065, USA; nagavendra.kommineni@gmail.com; 3 Department of Chemical Engineering, University of Benin, Benin City 1154, Nigeria; samuel.ebhodaghe@eng.uniben.edu; 4 Department of Pharmaceutical Sciences, Florida A&M University, Tallahassee, FL 32307, USA; bulusuraviteja@gmail.com; 5 Department of Pharmaceutics, National Institute of Pharmaceutical Education and Research, Hyderabad 500037, India; satyajuothi949@gmail.com; 6 Zoology Department, Faculty of Science, Cairo University, Giza 12613, Egypt; amanyasayed@sci.cu.edu.eg; 7 Department of Biology, Faculty of Science, University College of Taymaa, University of Tabuk, Tabuk 71491, Saudi Arabia; aawaji@ut.edu.sa; 8 Biology Department, College of Science, Jouf University, P.O. Box 2014, Sakaka 72388, Saudi Arabia; mogermoush@ju.edu.sa; 9 Regional Drug Information Center, Ministry of Health, Jeddah 21589, Saudi Arabia; hamdan27@hotmail.com; 10 Department of Pharmacology and Toxicology, Faculty of Pharmacy, King Abdulaziz University, Jeddah 21589, Saudi Arabia; mnasrullah@kau.edu.sa; 11 Department of Global Medical Science, Wonju College of Medicine, Yonsei University, Wonju 26426, Korea; 12 Department of Pharmaceutical Sciences, Pharmacy Program, Batterjee Medical College, P.O. Box 6231, Jeddah 21442, Saudi Arabia; 13 Pharmacology Department, Faculty of Veterinary Medicine, Suez Canal University, Ismailia 41522, Egypt

**Keywords:** microneedles, sustained and controlled release, transdermal drug delivery, natural polysaccharide

## Abstract

In this focused progress review, the most widely accepted methods of transdermal drug delivery are hypodermic needles, transdermal patches and topical creams. However, microneedles (MNs) (or microneedle arrays) are low-invasive 3D biomedical constructs that bypass the skin barrier and produce systemic and localized pharmacological effects. In the past, biomaterials such as carbohydrates, due to their physicochemical properties, have been extensively used to manufacture microneedles (MNs). Due to their wide range of functional groups, carbohydrates enable the design and development of tunable properties and functionalities. In recent years, numerous microneedle products have emerged on the market, although much research needs to be undertaken to overcome the various challenges before the successful introduction of microneedles into the market. As a result, carbohydrate-based microarrays have a high potential to achieve a future step in sensing, drug delivery, and biologics restitution. In this review, a comprehensive overview of carbohydrates such as hyaluronic acid, chitin, chitosan, chondroitin sulfate, cellulose and starch is discussed systematically. It also discusses the various drug delivery strategies and mechanical properties of biomaterial-based MNs, the progress made so far in the clinical translation of carbohydrate-based MNs, and the promotional opportunities for their commercialization. In conclusion, the article summarizes the future perspectives of carbohydrate-based MNs, which are considered as the new class of topical drug delivery systems.

## 1. Introduction

Polysaccharides are a class of biopolymers that influences the biological functions of living organisms, including structural support, energy storage, lubrication, and cell signal transduction [1,2]. Following recent discoveries on the novel role of biopolymers in medicine and pharmacy [3], the use of natural polysaccharides for pharmaceutical applications is now commonplace [4]. As a result, natural polysaccharides are important biomaterials that enhance the quality of healthcare services provided globally. This is because polysaccharide-based pectin [5] inhibits cancer cells, and the extracts from Grateloupialongifolia, Gracilarialemaneiformis, and others also inhibit the growth and activities of these cells [6,7]. Therefore, the use of polysaccharides for pharmaceutical applications addresses prevalent disease conditions such as cancer, which has caused 8.2 million deaths in recent years [8,9].

More recently, research has been increasingly reported on the use of microneedles (MN) for several pharmaceutical applications. This is due to their merit and potential. First, when used for transdermal drug delivery, they are capable of penetrating directly through the skin to the deeper layers of the dermis. This implies that they are able to deliver drug molecules across the skin [10,11] and maintain the local drug concentration for a long time at minimal invasive drug delivery [12]. Subsequently, MN array (MNA)-based drug delivery can ensure the local availability of therapeutics in chronic wound microenvironments, which is due to their potential in addressing physiochemical barriers usually present in the wounds [13]. More so, the MNA has been reported to be able to treat the complex pathophysiological nature of chronic wounds, particularly in microenvironments that require flexible delivery systems [14,15,16]. Finally, MNA can be used for the production of neocartilage tissue constructs for tissue engineering applications. Considering these merits, MNAs can be utilized for the printing of tissues [17], taking into account the role of the building blocks of cellular micro-spheroids. In bioprinting, MNA possesses the inherent capability of ensuring a sustained micro-spheroids orientation as well as proximity in culture. This is because the MNA often acts as a transient scaffold system for the application [18]. Therefore, microneedles (MNs) accelerate inflammatory inhibition, tissue formation, and several other applications when integrated with other biopolymers such as chitosan. In addition to tissue engineering and chronic wound healing, there is a need to review other pharmaceutical applications of MN-based natural polysaccharides, as most recent review articles on MNs have focused on their drug delivery potential.

Furthermore, MN is currently presented as a smart approach in enhancing transdermal drug delivery capabilities [19]. This review presents an overview of MN in their different forms, for instance, solid, hydrogel-forming, and hollow-types, with their potential and opportunities for extended application. The characteristics, advantages and applications of these MN types are also provided [20]. Although the use of MN for transdermal drug delivery has been signaled extensively, the role of technological advances in improving the research has been investigated. For example, Jung and Jin [21] presented a critical perspective on the use of digital technology in their review on current trends in MN for transdermal drug delivery. Building on the work of Waghule et al. [19], they provided an overview of the design, fabrication materials and methods for manufacturing MN. Up to now, research has been examining specific materials for fabricating MN [22]. Extending the scope of these reviews, Guillot et al. [23] reviewed strategies, pharmaceutical formulations, safety considerations, and applications of molecules in transdermal deliveries. Similarly, present studies have extended the research on different MNs types to include the components of critical therapeutic substances through the skin [24]. Therefore, advancing the review on several of these substances, including polysaccharides biomaterials, a more recent study has utilized the PRISMA guidelines to review the same for medical applications [25,26]. This shows that the goal of achieving clinical translation from the bench is in progress.

For this reason, we are providing an updated review on microneedle-based natural polysaccharides for pharmaceutical applications. Furthermore, MN characteristics and geometry, more comprehensive fabrication processes and future perspectives are presented in this review.

## 2. Microneedle and Materials

### 2.1. Microneedle Systems

The microneedle delivery system follows the diffusion mechanism to deliver the drug through a topical route by disrupting the surface layer of the skin temporarily. An array of hundreds of microneedles is arranged on a tiny patch, which aids in delivering enough drug that produces a therapeutic effect [19,21]. The dimensions of the needle must be optimized and confined to a limit as that of the thickness of the skin’s epidermis. If the needles are too long and thick, they can damage the nerves in the dermis region and cause pain and discomfort [27]. The thickness of the skin epidermis is 50 to 100 µm in general and specific areas of palms and soles are up to 1500 µm. Usually, these microneedles have a sharp tip with a length of 150–1500 µm, a width of 50–250 µm and tip thickness of 1–25 µm [19]. Microneedle tips can be of different shapes, for instance, pointed, pentagonal, cylindrical, triangular, octagonal and many more [24]. There are various microneedle designs depending on the fabrication method, delivery method, type of microneedle and drugs that are to be delivered. Different microneedles are prepared using different materials depending on the fabrication technique that was employed for the preparation of the microneedle. Next, we discuss a few properties of the materials.

#### 2.1.1. Silicon

Silicon has a crystalline structure and exhibits an anisotropic nature. The first ever microneedle prepared using silicon material was reported in 1990. The main benefit of the material is its flexibility in processing and production in the desired shapes and sizes [15]. This material provides a considerable mechanical strength that facilitates the disruption of the skin and delivers the drug at the site [28]. Successfully accomplished solid silicon microneedles have an average height of 158 m, a base width of 110.5 m, an aspect ratio of 1.43, a tip angle of 19.4°, and a tip diameter of 0.40 m. The mechanical stability of the constructed microneedles was evaluated by the Vickers hardness test and met the standards (solid silicon microneedles for drug delivery applications).

#### 2.1.2. Metals

Metals have had a great impact on the medical field for decades. The important metals that are used for microneedle production are stainless steel, titanium, palladium, nickel and palladium–cobalt alloys [14]. These materials have acceptable mechanical and biocompatibility properties. Metals are preferred over silicon-based microneedles because of their tough nature [19]. They developed a metal-based microneedle for a dry drug. The tip radius, height, and diameter at the middle section of the microneedle were 20, 467.8 and 268 μm, respectively, and the force required to break the skin to perform its action was tested on rabbit skin and confirmed to be 0.4 N [29].

#### 2.1.3. Ceramic

Ceramics have been used to produce microneedles primarily through a micro-mold technique. By using this technique, we can reduce the cost of the scale-up process. Alumina (Al_2_O_3_) is the main metal oxide that is used as it exhibits chemical resistance, forms stable oxides owing to its high energetic bonds between Al and O atoms and also remains unaffected by any environmental changes and corrosion. Other than this metal, Gypsum and Brushite are considered as they have been using these as drug-delivering means to bones [30]. S. Bystrova et al. introduced a micro-molding method to engineer a ceramic microneedle and achieved satisfactory mechanical performance [31].

#### 2.1.4. Silica Glass

Glass microneedles can be produced instantly with desired shapes and sizes. This glass metal allows ease while imaging fluid flow and fabricating microneedles, which are physiologically inert [15]. Though silica glass is used as an alternative to prepare microneedles, the usage is confined to laboratory purposes [32] and is not viable for commercialization. 

#### 2.1.5. Carbohydrate

Using the templates of silicon and metal microneedle templates, carbohydrate microneedles are prepared by molding with the hot melt method [33]. These are good alternatives toother microneedle materials because they are cost-effective, and importantly, they are safe for human health [34]. Maltose is the common sugar used to prepare microneedles [34]. Although they have some advantages, they also present a range of disadvantages. In some studies, they mentioned the inherent problems underlining processing to storage [33]. Ezgi P. Yalcintas et al. performed apoptosis and cell viability investigations on different carbohydrates (HA, CMC, trehalose, glucose, and maltodextrin) by constructing dissolving microneedles and confirmed that except for the high concentration of glucose, the rest of the carbohydrates are safe in engineering microneedles [35].

#### 2.1.6. Polymers

Polymers are under the spotlight because they exhibit better biocompatibility, biodegradability, minimal toxicity, and cost-effective materials for microneedle production. Usually, they are weaker than the above-mentioned materials but exhibit excellent toughness compared to ceramics and glass [36]. Polysaccharides are also used to prepare macromolecular dissolving microneedle systems. Xiaoyun Hong et al. and others reported that carboxymethyl cellulose, amylopectin, dextrin, hydroxypropyl cellulose, alginate and hyaluronic acid are commonly used materials to prepare macromolecular microneedles [36]. PVA was used in dissolving microneedles that increased the permeation of doxorubicin [37]. PEG- PMVE was used in hydrogel microneedle preparation to evaluate the antimicrobial activity [38]. Ethylene glycol was used in the preparation of a hydrogel-forming microneedle using a molding technique and achieved the sustained release of metformin HCl [39]. Ryan F. Donnelly et al. used the Gantrez polymer in optimizing and designing a polymeric microneedle using a laser-based technique [40].

### 2.2. Characteristics and Geometry of Microneedles

The geometry and characteristic properties of microneedles are very crucial during the design and preparation. The geometry of a microneedle, including its length and width, density of the array, shape of the needle, design of the needle, nature of fabricating material and fabrication process, is to be considered while preparing microneedles. Apart from that, the microneedle must have adequate strength to penetrate the skin and deliver the drug molecule or biomolecules into the skin. Penetration of microneedles across the skin must be ensured to avoid touching the nerves making it a painless application. Permeation of microneedles is affected by the length, shape, array density and width of the needle and its fabricated material [41]. However, the elastic nature of human skin can also resist the penetration of the microneedle into the skin, leading to twisting or breakage of the needle during its application, especially in the case of short and blunt needles [42]. The typical length of a microneedle varies from 150 to 1500 μm with a 50–250 μm width and diameter of 1–25 μm [43]. 

The microneedle design varies based on the process of fabrication, where they are categorized as in-plane and out-of-plane microneedles. In the case of in-plane microneedles, the longitudinal axis of the shaft is parallel to the surface of the substrate, whereas microneedles with out-of-plane have the longitudinal axis perpendicular to the surface of the substrate. The in-plane design of microneedles allows regulated tailoring of the needle length, which is considered as its major advantage. However, it encounters difficulty in the process of its preparation. Out-of-plane microneedles facilitate a gentle fabrication process with a high-density array of microneedles. Nevertheless, out-of-plane design may hamper the control of the needle length and aspect ratio, resulting in shorter height and a lower aspect ratio [44,45]. The tip of the needle also has various shapes where the most widely used shapes are pyramidal, cylindrical, conical, rectangular, octagonal, and quadrangular. The shape of the needle tip affects the penetrating potential of the needle, where the tip with pyramidal shape has better mechanical properties compared to the conical-shaped needles. This is due to the high cross-sectional area of the pyramidal-shaped needle having the same base width [46]. The penetration of microneedle is also affected by its shape where sharper and narrower tips require low application force and needle tips with large diameter require larger application force on the needle to penetrate into the skin [47]. 

**Figure 1:**
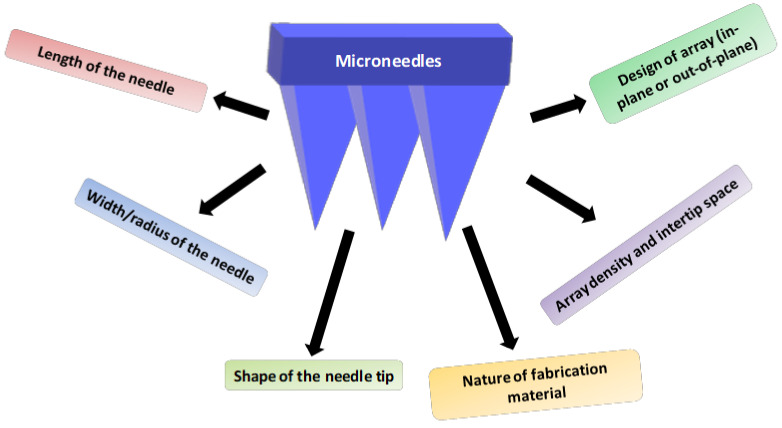
Characteristic features to be considered during the preparation of microneedles.

Moreover, the length of the tip and the inter-tip spacing also affect the penetrating potential of the microneedle where needles with longer length, wider width and a high density array will result in greater penetration into the skin [48]. A study was performed to assess the effect of the radius of the tip on the penetration depth. Chitosan microneedles with a 10-µm tip radius resulted in deeper penetration into the skin compared to the needle with a 5 µm radius [49]. An amalgamation of all these characteristic features is to be controlled to achieve the overall success of microneedle penetration and delivery of drugs and biomolecules into and across the skin (Figure 1).

### 2.3. Fabrication Techniques

These techniques are of different types. The choice of techniques depends on the type of drug that is incorporated in the microneedle, dose, desirable pharmacokinetics and pharmacodynamics, targets and design or material.

#### 2.3.1. Laser Cutting

This technique is used to manufacture metal or polymer-based microneedles. Out of all, the most used metal is stainlesssteel [42,50,51]. This laser machine is connected to a computer-based software called Computer-Aided Design (CAD), which assists in designing the microneedle size and orientation [21]. The 2D shape of the microneedle is created using a laser to cut a metallic sheet. This created 2D design is used to create a 3D design by bending the angle to 90°. The created needles or ridges on the flat metallic surface are cleaned through electropolishing [52,53].

#### 2.3.2. Laser Ablation

This is another laser-mediated technique that is also used to manufacture metal or polymer-based microneedles. Unlike the laser cutting method, it creates and engraves the plate into 3D microneedle plates [21,54]. For instance, when the CO_2_ laser beam is irradiated on the substrate, it absorbs heat energy and undergoes either evaporation or sublimation. By using this process, we can extract an inverse mold by generating a microneedle pattern [21].

#### 2.3.3. Photolithography

This technique is used extensively to produce hollow or solid microneedles. Based on the microneedle structure, an inverse mold method is employed to manufacture the silicon or dissolve hydrogel microneedles. The process for manufacturing silicon microneedles is a thin sacrificial layer deposited on the pre-treated silicon on which a photoresist, a photosensitive polymer, is coated using a spin coating technique [21]. Then, the subsequent process of developing tips utilizes different types of etching processes, e.g., dry etching and wet etching.

#### 2.3.4. Etching

When the microneedle is prepared by using photolithography, etching remains the most crucial step as it defines the shape of the microneedle tip. The size of the microneedle base and the gap between the microneedles are determined before the etching process [55]. It is classified into two types, wet and dry etching, which results in isotropic or anisotropic etching depending on the method employed.

#### 2.3.5. Dry Etching

This method is used primarily to manufacture solid or hollow microneedles by two methods—physical and chemical methods [10,21,56]. The physical method includes ion milling and sputtering, whereas the chemical method includes high-pressure plasma etching. In dry etching, by using high-energy and unidirectional electrodes, an inert gas becomesionized, and these energized ions strike the silicon substrate to create an anisotropic effect. In the physical manufacturing process, if the substrate or sacrificial layer is protected using oxide film or photoresist, then that part is barely etched, and on the other hand, the part without the photoresist layer is etched.

In the chemical process, the gas plasma becomes chemically active and reacts with the surface of the substrate and converts it into a volatile substance, thereby producing an isotropic etching effect [16]. However, a combination of the above-mentioned methods can be employed to control the isotropic and anisotropic etching. Watchful optimization can deliver a precise microneedle tip [57].

#### 2.3.6. Wet Etching

This process is used to fabricate metal and silicon microneedles by using a chemical etchant and produces a pattern of events on the substrate [58]. For instance, this method is employed in producing a silicon wafer in which a potassium hydroxide solution is used as a chemical etchant [57]. The shape of the microneedle tip can be modified by altering the etching rate, and it depends upon the direction of silicon crystals. The etching rate is significantly faster than dry etching, and this process also follows isotropic etching similar to dry etching. The main limitation of this process is its poor accuracy for fine fabrication.

#### 2.3.7. Three-Dimensional Printing

Three-dimensional printing has expanded its wings to various fields, which includes the manufacturing of microneedles. This technology helps in producing not only simple microneedle structures but also complicated structures without compromising accuracy. There are a few different high precision techniques [59,60].

#### 2.3.8. Micro-Stereolithography

This method was introduced in the late 1980s [60,61]. Since then, it has been used in various fields, primarily in biomedical and tissue engineering. By using this technology, currently, researchers are manufacturing tissue scaffolds, nerve guidance conduits and cardiovascular stents [62]. This procedure is reliable and capable of producing high complex microneedles with great precision. These 3D objects are generated by controlled solidification of liquid resin using photopolymerization (UV radiation). This solidification helps the resin to adhere to the support platform, and the built layer is recoated with liquid resin. Thus, this process is also called a layer-by-layer fabrication process [63].

#### 2.3.9. Continuous Liquid Phase Production

This process also comes under the layer-by-layer approach. Continuous liquid phase production is a traditional system that fabricates an object by photopolymerization of resin using a digital light process. The working principle of continuous liquid interface production is similar to that of digital light. Through this system, we could be able to produce a microneedle within 10 min by eliminating the rate-limiting steps that conventional methods face during development [64]. Some papers have reported the usage of biocompatible polymers in the production of microneedles [65].

#### 2.3.10. Two-Photon Polymerization

This method is a complete additive manufacturing process that can be used to produce microneedles of approximately 100 nm [66]. Unlike the continuous liquid interface production technique, this system employs a near-infrared wave-length laser instead of UV radiation. This laser initiates polymerization of the resin by multiphoton absorption. It can produce extensive and complex 3D designs [67,68].

### 2.4. Mechanical Properties of Natural Microneedles

In numerous microneedle studies, the mechanical strength of the microneedle patch has been examined by compressing the entire patch against a flat surface, and then the rupture force of individual microneedles has been calculated by taking the total rupture force and dividing by the number of needles. This strategy is inadequate because it does not identify possible variations in mechanical properties between microneedles across the patch [69]. The mechanical properties obtained are also limited to the breaking strength of the overall patch. In other studies of microneedles, their mechanical properties were not measured directly but were reflected by their efficiency of penetration into the skin. The small holes in the skin generated by microneedle penetration were normally stained and visualized to calculate the skin penetration efficiency. However, this method does not provide any quantitative results on the mechanical properties of the microneedles. Atomic force microscopy (AFM) was also used to measure the mechanical properties of microneedles, and the depth or force of penetration is limited to a nanoscale measurement [69].

Yuquan Chi et al. developed the fabrication of three types of HA-MNs with different molecular weights (10, 74 and 290 kDa), which incorporate rhodamine B as a model drug. We evaluate the influence of HA molecular weight on the mechanical properties of HA-MNs and the transdermal delivery of rhodamine B in vitro and in vivo. The mechanical strength of all types of HA-MNs exceeds the minimum force required for skin penetration, with the highest values of compressive force found at 10 k HA-MN. Interestingly, 74 k HA-MN, which has medium mechanical strength, exhibits the highest efficacy in transdermal delivery of rhodamine B in transdermal pigskin and Franz cell model [70].

### 2.5. Advantages of Natural Microneedles

Recently, natural microneedles for transdermal drug delivery have received increasing attention as they can provide painless, minimally invasive drug delivery [71].

It was in the early 19th century that methods of enhancing skin transport for transdermal delivery received enormous interest to conduct extensive research. As is well known, transdermal drug delivery through the skin faces a major challenge due to the strong barrier of the intact stratum corneum. Transdermal delivery has many significant advantages over intramuscular injection, subcutaneous injection and intradermal injection. Therefore, the microneedle is considered an effective and painless device, easy to use for patients, promising for the delivery of macromolecules in the field of transdermal drug delivery. However, Jian Yang et al. discussed recent advances of microneedles for biomedical applications: drug delivery and beyond [72].

## 3. Types of Microneedles

There are numerous types of microneedles that have been classified depending upon the fabrication and their desired application, including solid, coated, dissolving, hollow, and hydrogel-forming MN. Each type of microneedle has its own benefits and limitation for delivery of the drug at the target site. However, some researchers classified microneedles based on the fabrication method [73]. As the characteristics of microneedles vary by type, an appropriate microneedle design should be selected based on drug dose, the onset of action, delivery period, delivery efficiency, packaging, cutting waste, and patch wear time (Table 1 and Table 2).

### 3.1. Solid Microneedles

**Figure 2:**
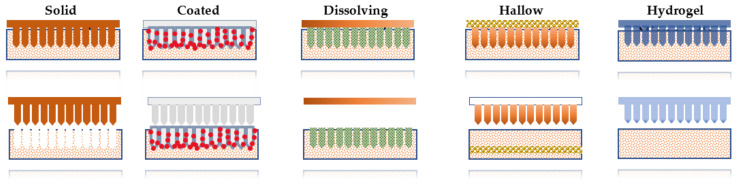
Schematic representation of drug flow of different microneedles.

A solid microneedle is generally used to deliver the drug through micronized channels formed in the skin layer to increase the permeability of the drug molecules [21]. In this type of microneedle, the drug molecule is attached to the channel, and by the time of termination of the therapy, the microchannel needs to be closed to avoid the unnecessary entry of the toxic substance through the microchannel. Solid microneedles can act as a drug reservoir. There are various non–biodegradable metals used for the fabrication of solid microneedles (Figure 2).

These MNs are fabricated with the pointed tips at the end, which facilitates micronized pore creation on the epidermal surface of the skin [74]. In general, this microneedle permeates the drug molecule through the passive diffusion mechanism. To date, various materials have been applied to the fabrication of solid microneedles, including biodegradable and non-biodegradable, such as silicon, and polymers, including methyl vinyl ether and maleic anhydride (PMVE/MA), polycarbonate, polymethylmethacrylate (PMMA), maltose, stainless steel, titanium, and nickel, etc. Conventionally, micropumps and microreactors are being used for the fabrication. Drug delivery through solid microneedles is influenced by various factors, including MN insertion force, tip sharpness and MN density. However, the fabrication of solid microneedles can be achieved by microfabrication technology, i.e., micro-machining or micro-electromechanical systems (MEMS) [22].

**Table 2:** Type of microneedle system.

Microneedle	Material	Technique Employed	Approach	Type of Product	Improvements	References
Solid Microneedles	Silicon	Dry and Wet Etching	Poke and Patch	Docetaxel Liposomes	Skin permeation	[75]
	Derma-roller	NA		Topical 5-FU	Invitro and in vivo anti-tumor activity	[76]
	MNs coated with ZnONanowires	Photolithography		Paclitaxel	10% increase in reduction of tumor size compared to conventional method	[77]
	Stainless Steel	-		Combinational (Mesoporous Nano Particles) Therapy of Phthalocyanine, Dabrafenib, Trametinib	Inhibited cell proliferation and anti-tumor activity by reactive oxygen species	[78]
Coated Microneedles	Stainless Steel	Infrared Laser Cutting, Ink-jet Printing	Coat and Poke	5-FU, Curcumin and Cisplatin	Ink-jet printing on SS Microneedles	[79]
	Stainless Steel	Wet Etching		PLGA Nanoparticles of DOX	Effective local delivery for oral cancer	[80]
	Stainless Steel	Manual Coating		Octa-Arginine siRNA Nanocomplexes	Induced BRAF gene, which is responsible for melanoma development, induce tumor apoptosis and proliferation	[81]
	Polycarbonate	Dip Coating		Immunotherapy using DNA Polyplexes and Poly Adjuvant	Induced humoral and cellular immunity facilitated targeting and activation of skin	[82]
Hallow Microneedles	Nickel	-	Poke and Flow	DOX	Increased drug diffusion coefficient	[83]
	Stainless Steel	-		5-FU	Effective against gastric cancer cells	[84]
	Silicon	Manual Coating		HPV 16 E6 siRNA	Targeted delivery and inhibited tumor progression and observed no major adverse reactions	[85]
Dissolving Microneedles	Polyvinyl Alcohol (PVA)	Micro Molding	Poke and Dissolve	DOX	Improved permeation	[37]
	Zein	Micro Molding		Tamoxifen and Gemcitabine	No improvement for tamoxifen, observed great permeation in gemcitabine	[86]
	Sodium CMC	Micro Molding		Lipid-XoatedCisplatin Nanoparticles	Enhanced cytotoxicity and reduced tumor size	[87]
	Pluronic F127	Micro Molding		Cancer Vaccination for EG7-OVA Tumor	Improved antigen-specific humoral and cellular immunity	[88]
Hydrogel Microneedles	PLGA	Multiple Casting	Poke and Swell	Amphotericin	Controlled, prolonged release of drug for a week	[37,89]
	Ethylene Glycol Methylvinylether-co-maleic acid	Molding		Metformin HCl	Sustained release	[39]
	PEG-PMVE/MA	Micro Molding		Anti-Microbial	No microbial invasion through skin	[90]

### 3.2. Dissolving Microneedle

These MNs are based on the principle of the poke and release approach. Dissolving microneedles are usually made up of biodegradable polymers including poly (propylene), dextrin, chondroitin sulfate, and albumin, polylactic acid (PLA), polyglycolic acid (PGA), polylactic-co-glycolic acid (PLGA), polyvinylpyrrolidone (PVP), poly (vinylpyrrolidone co-methacrylic acid) (PVPMAA) and poly (methyl vinyl ether-maleic anhydride) (PMVE-MA). These microneedles are preferred over other types of microneedles due to their own promising characteristics of self-dissolving and lower risk of associated toxicity. The first report on dissolving MNs was reported in 2005 by Miyano et al. [34]. In the manufacturing of dissolving microneedles, polymer selection is a crucial step, which needs to be taken into consideration as it affects the release kinetics of the drug (Figure 2).

In the literature, there are various examples reported for the application of dissolving microneedles for synergistic drug delivery in which other techniques are used along with microneedle drug delivery [91]. The application of dissolving microneedle is not limited to the drug incorporation but has also enhanced the preparation of the microneedle patch for the vaccine delivery against influenza, adenovirus vector, etc. (Table 2). Dissolving microneedles can be prepared using various methods, including solvent casting, laser machining, droplet-born air blowing, microinjection molding, hot embossing, ultrasonic welding and lithography. The solvent casting method is widely used in the fabrication of dissolving microneedles [92]. Solvent casting can be performed by the ultrasonic welding method in which polymers are fused without heat. While encapsulated drugs are controllably released after penetrating into the skin, dissolving MNs have poor mechanical properties due to their high hygroscopicity. 

### 3.3. Coated Microneedle

Coated microneedles are covered by the drug solution or dispersion coat, which serves a rapid and bolus release of drug molecules. There are various reports available where DNA, protein and peptide delivery weresuccessfully carried out with a coated microneedle approach [93]. Coating of the drug on the surface of the microneedle enables the drug dissolution into the skin. It has been demonstrated for the siRNA incorporation with the minimally invasive method [94].

There are some parameters that need to be taken into consideration, including the homogenous coating on the surface of microneedles and controlled and precise drug release from the fabricated microneedle (Table 2). These microneedles possess the long-term stability of active ingredients. Various methods have been employed in order to coat the solid microneedle, including dip coating and spray coating. In spray coating, the droplet is fully covered on the surface of the MN, and this serves as a more scalable method for the fabrication of coated microneedles (Figure 2). 

Gill and Prausnitz highlighted that a reduction in surface area and increase in viscosity could improve the efficiency of the coating microneedle [95]. In addition, layer coating is also reported where the MN is immersed in an oppositely charged solution, such as negatively charged DNA that interacts with a positively charged polymer. Piezoelectric inject printing is also reported for the deposition of antifungal drug coating on the surface of microneedles [96].

### 3.4. Hydrogel Forming Microneedle

Hydrogel-forming cross-linked polymers are largely influenced by molecular weight, swelling index and the presence of a foaming agent. There are various parameters that affect the delivery of drugs through a transdermal route. In this type of microneedle, there is no restriction on the incorporation of various types of drugs (Table 2). This strategy was first established by Donnelly et al., who used a super swellable polymer in the microneedle infrastructure [97]. The array contains no drug as such, but upon penetration, it imbibes through the skin layer. The range of fluid withdrawal in 1 h was 0.9 to 2.7 μL, which is of the same order of magnitude as the interstitial fluid withdrawal rates for hollow MNs and microdialysis.

The limitation associated with conventional microarray techniques can be overcome by the hydrogel MN, which includes reducing drug loading capacity, control of the extent of the release and precise drug coating. It serves potential tuning benefits in which the desired shape and size of hydrogel-loaded microneedle (Figure 2) can be fabricated, which can be sterilized easily [45]. A hydrogel-based microneedle is a versatile device in which various drugs with different therapeutic windows can be loaded into the hydrogel for personalized treatment options [98]. This microneedle therapy is used for the sustained release of metformin HCl for 24 h and is also used to monitor or quantify drug substances [39,99].

### 3.5. Hollow Microneedles

This microneedle differs from other types of microneedles in terms of length and diameter of its structure. It is prepared with a 30 gauge hypodermic needle where the specific microneedle has a length of 300 micrometers. Numerous materials are mainly used in the fabrication of these microneedles, which include silicone, glass, ceramic and polymer, etc. This system can deliver the drug rapidly by passive diffusion compared to the other types of microneedles. In addition, other stimuli such as pressure and electrically driven transport are also feasible in this class of microneedle [15]. It has been reported that various parameters affect the flow rate of the drug through hollow microneedles such as the inner diameter of MN, tip dimension, pressure, insertion and retraction depth and length of microneedle (Table 2). Multiple techniques are available for the fabrication of hollow microneedles, including the MEMS techniques, profound engraving of silicon by reactive ions, an incorporated lithographic molding technology, advanced X-ray photolithography and wet chemical printing and microfabrication. In recent years, hollow microneedles have been employed to be fabricated by 3D printing methods (Figure 2).

## 4. Mechanism of Drug Delivery with Microneedles

**Figure 3:**
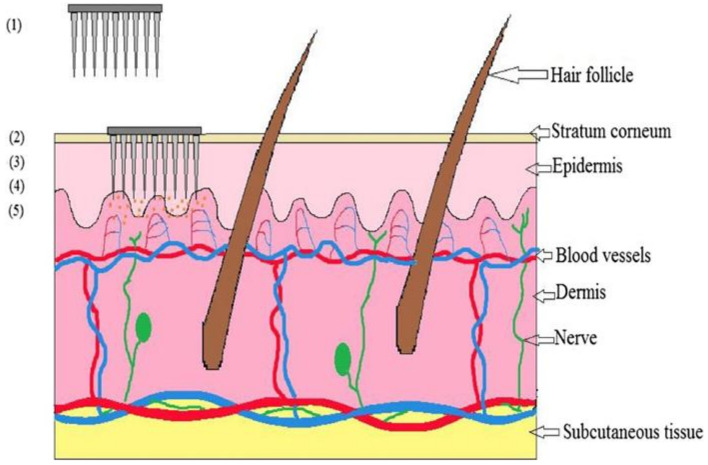
Mechanism of drug delivery by microneedle device: (1) microneedle device with drug solution; (2) device inserted into the skin; (3) temporary mechanical disruption of the skin; (4) release of drug into the epidermis; (5) transport of drug to the site of action. Reprinted with permission from Ref. [19]. Copyright 2019 Elsevier.

The administration of topical drugs depends on the diffusion mechanism. In the microneedle drug delivery system, the skin is briefly interrupted [100]. A microneedle device is made by placing thousands of microneedles in an arrangement on a tiny patch (identical to a normal commercially available transdermal patch) to deliver sufficient amounts of a drug to obtain the necessary therapeutic response. It punctures the stratum corneum, bypassing the barrier layer. The drug is delivered directly into the epidermis or uppermost layer of the dermis, then passes into the systemic circulation and exhibits a therapeutic response when it reaches the site of action [101]. The mechanism of drug delivery by microneedles is demonstrated in Figure 3.

## 5. Natural Polysaccharides Used in Microneedles

**Figure 4:**
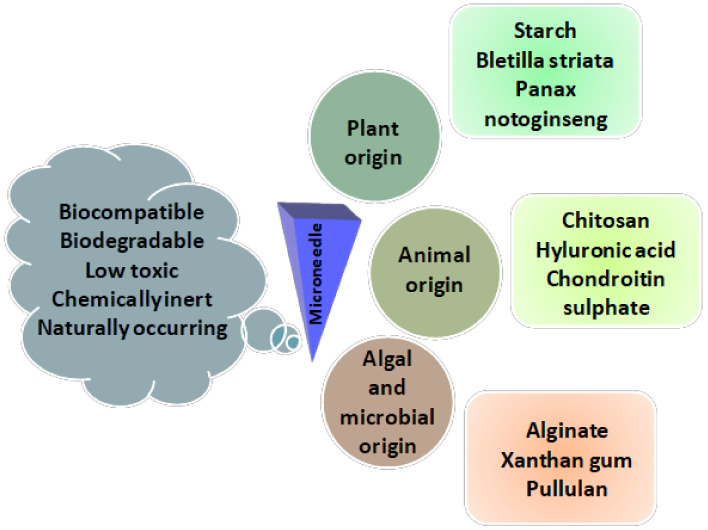
Advantages and sources of natural polysaccharides used in the fabrication of microneedles.

Polysaccharides have been widely explored in the field of drug delivery systems due to their biocompatibility, biodegradability and low toxicity [102,103]. Exploitation is also taking place in the fabrication of microneedles [104]. There are various polymers used in transdermal drug delivery due to their physiochemical properties and tenability and mechanism of drug release. Polysaccharides are obtained from natural sources such as plants, animals, and microorganisms, etc. However, polysaccharides are extensively used due to their biocompatibility, biodegradable nature, ease of fabrication and sustainable delivery. The most widely explored polysaccharides are not limited to hyaluronic acid (HA), dextran, chitosan (CS), cellulose, sodium alginate (SA) and blends of other biopolymers [105]. They are more advantageous than synthetic polymers pertaining to environmental friendliness (Figure 4).

### 5.1. Hyaluronic Acid (HA)-Based MNs

Hyaluronic acid (HA), also called hyaluronan, is a polysaccharide composed of d-glucuronic acid and N-acetyl-d-glucosamine, which are linked by β-(1,4) glycosidic bonds. It is a simple, water-soluble polysaccharide [106]. It is widely present in the skin and synovial fluid joints [107]. However, it is extracted from rooster combs, shark skin and microorganisms. It was approved by USFDA for its use in soft tissue damage. It is used to fabricate dissolving microneedles, which dissolve after penetrating into the skin and release the drug (Table 3). It also accommodates high amounts of a drug, leading to superior delivery and quicker onset of action [108]. A study aimed to design insulin delivery via hyaluronic acid microneedles resulted in the complete dissolution of microneedles into the rat skin after 1 h of application. This showed the self-dissolving ability into the skin and release of loaded molecules to the targeted site. The plasma peak levels were also compared with microneedles and subcutaneous injection, where a higher level of insulin was achieved with microneedles. This study highlighted the potential of hyaluronic acid in designing dissolving microneedles for transdermal drug delivery [109]. Hyaluronic acid has also been shown to have fine mechanical properties that penetrate the thickened epidermis of mice induced with psoriasis. The dissolving property of hyaluronic acid microneedles exhibited superior efficacy compared to the oral delivery in treating psoriasis, highlighting the dissolving microneedle patch as an excellent strategy for the effective delivery of the drug [110]. Dissolving microneedles tailored with hyaluronic acid were also investigated for delivering high molecular weight drug molecules. A high molecular weight of 4000 Da fluorescein isothiocyanate-labeled dextran was used and assessed for its permeability and accumulation in the skin. Transcutaneous electrical resistance and trans-epidermal water loss were found to be increased, revealing the penetrating ability of hyaluronic acid microneedles [111].

Jinjin Zhu et al. demonstrated that 5-Aminolevulinic acid (5-ALA), which is an endogenous nonprotein amino-acid-loaded HA microneedle, was effective for pharmacodynamics therapy in the superficial tumor treatment. The results of this study revealed that 5-ALA effectively reached the target site by penetrating the stratum corneum upon administration by the HA microneedle. In addition, this HA microneedle showed superior long-term stability and activity at room temperature due to the acidic and oxygen-free environment of HA MNs [112]. In another study, hyaluronic acid was used as a matrix for the fabrication of a microneedle patch in order to incorporate a synergistic combination of gene therapy and photothermal therapy for cancer treatment. The p53 DNA and IR820 were co-loaded in a HA microneedle patch, and the result showed that the fabricated microneedle efficiently penetrated through the stratum corneum and could deliver the drug to a subcutaneous target site. It was concluded that this strategy could be seen as the best suitable alternative and act as a synergetic strategy [113]. Moreover, near-infrared sensitive dissolvable microneedles are reported for the treatment of human epidermoid cancer and melanoma. Thus, microneedles were fabricated by the HA and loaded with light-responsive 5-fluorouracil (5-Fu) and indocyanine green (ICG)-loaded monomethoxy-poly (ethylene glycol)-polycaprolactone (MPEG-PCL) nanoparticle (5-Fu-ICG-MPEG-PCL), and then 5-Fu-ICG-MPEG-PCL. Ying Hao et al. concluded in this study that an HA-based microneedle was proven to have good penetration ability and heat transfer efficacy. This also contributed to the controlled release of the incorporated drug and could successfully develop a synergistic treatment of chemotherapy and phototherapy for cancer [114].

Hongyao et al. prepared an HA-based microneedle for the treatment of psoriasis, which enables higher water solubility, biocompatibility, mechanical properties and biodegradability [110]. Microhyala is an FDA-approved product of microneedle used in the cosmetic market as it dissolves in intestinal fluid and is degraded by free radicals, which are found in the extracellular matrix and lysosomal enzymes [22]. In addition, IvySaha et al. compiled multiple applications of an HA MN array in the cosmetics and medical field. We recommend that the interested reader refer to the review paper, which will provide more knowledge on this specific topic [108]. There are various methods used for the preparation of HA microneedles, such as micro-molding, photopolymerization, and drawing lithography [115]. HA microneedles (MN) have been used to load various therapeutic molecules for its effective treatment.

### 5.2. Chondroitin Sulfate-Based MNs

Chondroitin sulfate is a disaccharide sugar moiety comprised of N-acetyl-galactosamine and d-glucuronic acid bonded by β-(1,3) glycosidic linkages. It is naturally available in cartilage, porcine skin and bovine trachea. Based on the source, marine or terrestrial animals, the composition and concentration of chondroitin sulfate varies [116]. It is a water-soluble saccharide that forms a dissolving microneedle [117]. A dissolving microneedle of recombinant staphylococcal enterotoxin B was designed with chondroitin sulfate and trehalose, which showed good penetration ability with 260 µm depth of penetration into the skin of mice (Table 3). This study showed effective immunization via chondroitin sulfate microneedle transcutaneously [118]. A two-layered dissolving microneedle was prepared with chondroitin sulfate and dextran individually as the base polymer for the delivery of recombinant human growth hormone and desmopressin. Chondroitin sulfate microneedles showed a higher extent of bioavailability as compared to the microneedles prepared with dextran [117].

### 5.3. Cellulose-Based MNs

Cellulose is a naturally occurring, abundant biomaterial composed of herbal cells and tissues. It is obtained from various sources, including wood, cotton, bacteria and algae. However, commercially, it is procured from wood and cotton. Cellulose contains glucose monomers, which are linked only by β-(1,4) linkages (Table 3). There are numerous cellulose derivatives used in the pharmaceutical sciences. Cellulose esters and cellulose ethers are widely used derivatives from the cellulose family. Cellulose esters are water-insoluble and film former polymers such as cellulose esters, including cellulose acetate, cellulose acetate phthalate and cellulose nitrate [119]. In the literature, one of the patents revealed the application of cellulose-based microneedles in cancer therapy [120]. Furthermore, selected inventors from the University of Pittsburgh and Carnegie Mellon University have divulged carboxymethylcellulose (CMC) MNs that can release a number of different chemotherapeutic agents and immune-stimulating agents that can be used either alone or in combination. In this example, doxorubicin, valrubicin, epirubicin, idarubicin and other known anthracycline agents have been utilized for the treatment of skin cancer [121]. Patent No CN106426729A highlighted that cellulose microneedles are not only used for gene delivery but are also applicable for the delivery of anti-cancerous agents [122,123]. Yong-Hun Park et al. fabricated a microneedle for transdermal delivery by laser writing and replica molding processes. A threefold enhancement in permeability was observed. The authors reported that this fabrication process was best suitable for a CMC microneedle, even in cosmetics products [124]. Daniela F.S. Fonseca et al. developed dissolvable hyaluronic acid (HA) microneedles (MNs) combined with bacterial nanocellulose (BC) MN patched for dermo-cosmetic application. HA- and BC-blended microneedles had sufficient mechanical strength, and BC contributed to a controlled release of the drug molecule. The in vivo safety studies also reported that the prepared microneedle was safe and biocompatible [125].

### 5.4. Chitin and Chitosan(CS)-Based MNs

Chitosan is a marine polysaccharide extracted from chitin [103,126,127]. Naturally, it is found in the cell wall of fungi. Chitosan is obtained from the deacetylation of chitin [103,126]. It is a linear biopolymer formed by d-glucosamine and N-acetyl-d-glucosamine linked by a β-(1,4) bond. It has a molecular weight in the field of 300 and 1000 kDa (Table 3). It has been observed that the low molecular weight polymer has poor mechanical strength and in order to improve it, PLGA was added [128]. Chitosan naturally possesses antibacterial and wound healing properties. It is a water-insoluble polymer and is degraded by lysozymes and chitosanase. André F. Moreira et al. demonstrated that the blend of polyvinyl alcohol and chitosan are used for manufacturing micro-needles [129]. Micro-molding and electro-spraying techniques were used in combination in the fabrication of this microneedle intended to deliver doxorubicin and AuMSSnanorods. The Dox@MicroN patches were observed to have good photothermal ability resulting in a temperature enhancement of 12 °C under near-infrared irradiation. Nevertheless, the microneedles were able to penetrate the tumor-mimicking agarose gel and promote layer-based drug release [129]. It is reported that the addition of the thiol group improves the mechanical properties of chitosan, and hence, thiolated MNs are prepared with optimum mechanical strength and sharpness [130].

Mei-Chin Chen et al. introduced chitosan microneedle patches for sustained drug delivery of hydrophilic drugs. It was reported that 95% in vitro drug release was obtained through this drug delivery within 8 days. In addition, the incorporated BSA molecule was diffused by a penetration depth of 300 μm [49]. Water-soluble chitosan is also prepared by treatment of trifluoroacetic acid followed by a 0.1 M NaCl solution. This method was found to be suitable for sustained transdermal drug delivery of more than 72 h [131].

### 5.5. Starch-Based Microneedle

Starch is a biodegradable material available naturally in extensive amounts for various properties and applications in the biomedical field (Table 3). It has been explored widely in formulation practices due to its brittleness and multipurpose applications. Yujie Zhang et al. demonstrated a new microneedle patch that disperses and releases insulin in response to glucose for type 1 diabetes. It was observed that a fabricated microneedle was complete and uniform in structure. In this, the nanomaterial was added as an additive to enhance the mechanical strength [132]. A starch and gelatin blend was also used for the fabrication of microneedles for the treatment of losartan through transdermal drug delivery [133]. Starch-based amylopectin is not biodegradable and hence not preferred for the fabrication of microneedles. Pablo Serrano-Castañeda et al. [134] highlighted microneedles as enhancers of drug absorption through the skin.

### 5.6. Sodium Alginate (SA)-Based Microneedle

Alginate is a natural polysaccharide obtained from algal and bacterial sources. The commercial source of alginate is brown algae. It is an anionic linear polymer consisting of α-l-guluronic acid and β-d-mannuronic acid saccharide units [135]. A study was conducted to determine the delivery potential of sodium alginate microneedles where bovine serum albumin was used as a model drug (Table 3). The results showed an improvement of 15.4 fold permeation compared to sodium alginate needle-free patches, thus availing the microneedle for transdermal delivery [136]. Another study reported the delivery of insulin with alginate and maltose composite as a base polymer for the preparation of a microneedle. The composite of alginate and maltose revealed higher mechanical strength for the penetration of microneedles into the rat skin as a transdermal drug delivery system [137].

GwenaëlBonfante et al. discussed three materials—carboxymethyl cellulose (CMC), alginate, and hyaluronic acid (HA)—for the manufacture of microneedles. However, the microneedles have been designed with low concentrations for rapid dissolution while maintaining the strengthening effect and were used varying from 1 to 5% (*w*/*w*) in deionized water [138]. Their overall performance aspects, such as geometric parameters (width, height, and tip width), piercing capabilities, and dissolution time, are measured and discussed. To break the skin barrier, two key parameters, a sharp tip and overall mechanical strength, are highlighted. Each material fails the piercing test at a concentration of 1% (*w*/*w*). Concentrations of 3% (*w*/*w*) and 5% (*w*/*w*) result in powerful matrices capable of piercing the skin. For the purposes of this study, HA at a concentration of 3% (*w*/*w*) results in arrays consisting of microneedles with a tip width of 48 ± 8 m and pierces through the sheet with a dissolution time of less than 2 min.

### 5.7. Xanthan Gum (XG)-Based Microneedle

Xanthan gum (XG) is a hetero polysaccharide with β-(1,4)-d-glucopyranose glucan as a backbone with (3,1)-α-linked d-mannopyranose-(2,1)-β-d-glucuronic acid-(4,1)-β-d-mannopyranose as side chains (Table 3). It is produced by bacteria called Xanthomonas campestris, where it is secreted from the surface of the cell wall by enzymatic reaction [1]. Xanthan gum is extensively used as a viscosity enhancer of the coating solution in the preparation of coated microneedles. A study was reported to use xanthan gum at 1% *w*/*v* concentration as a coating solution to the microneedle coated with an influenza virus-like particle vaccine where it showed good hemagglutinin activity as compared to carboxymethyl cellulose. However, carboxymethyl cellulose was superior in terms of coating dose than xanthan gum, and thus, carboxymethyl cellulose was used as a viscosity enhancer [139]. In another study, xanthan gum at 0.075 % *w*/*v* was used along with trehalose as a viscosity enhancer for the influenza vaccine coated microneedles. Nevertheless, hemagglutinin activity was significantly high as compared to the trehalose solution. These studies suggested further investigation of xanthan gum in the preparation of microneedles [140].

### 5.8. Pullulan-Based Microneedle

Pullulan is another hydrophilic polymer comprising three maltose saccharide units linked by α-1,6 glycosidic bonds, which is a trisaccharide sugar moiety. The source of pullulan is Aureobasidiumpullulans, which is a yeast-like fungus (Table 3). It possesses sufficient mechanical strength to fabricate microneedles [141]. A dissolving microneedle patch of pullulan was fabricated for the delivery of insulin by a micro-molding method. The microneedle showed a penetration depth of 381 µm into the skin and dissolved into the skin within 2h and released 87% of insulin [142]. The pullulan microneedle was also assessed for the delivery of small and large biomolecules across the skin. Ex-vivo skin permeation studies have been performed on porcine skin and concluded that pullulan dissolving microneedles have huge potential for transdermal drug delivery.

### 5.9. Bletilla Striata (BS)-Based Microneedle

Bletilla striatum isa herbal-based polysaccharide obtained from the tubers of Bletilla striata. It is broadly known for its medicinal properties, and it is a water-soluble polysaccharide comprising α-mannose, β-mannose and β-glucose as monosaccharide units [143,144,145]. A dissolving microneedle-based on Bletilla striata polysaccharide was prepared with rhodamine B as a model drug. The study showed promising results for its effectiveness in penetrating the skin and delivery of loaded moiety and was assessed by a texture analyzer. Confocal laser scanning microscopy signified the dissolution of the microneedle and the release of molecules into the skin. Thus, this study has highlighted the prospect of novel polysaccharides in tailoring dissolving microneedles [146]. Panax Noto ginseng is another herbal-based polysaccharide investigated for the preparation of a dissolving microneedle for transdermal drug delivery [147]. The prepared microneedles showed good mechanical properties and sufficient penetrating ability, demonstrating transdermal delivery of drugs across the skin [148]. Polysaccharides derived from natural sources are, therefore, extensively studied for the generation of microneedles, and the summary of the polysaccharides used is given in Table 3.

**Table 3:** Summary of polysaccharides used for the preparation of microneedles.

Polysaccharide	Source	Monosaccharide Units	Type of Microneedle Fabricated	Inference	Reference
Chitosan	Derived from chitin (natural sources of crustacean family)	d-glucosamineand N-acetyl-d-glucosamine	Hollow–solid, dissolving, and coated layer-by-layer microneedles	Possess good mechanical strength and also availed for its adjuvant and antibacterial property	[129,149,150,151]
Hyaluronic acid	Rooster combs, shark skin	d-glucuronic acid and N-acetyl-d-glucosamine	Hollow, dissolving and hydrogel microneedle	Self-dissolving ability and good penetration	[109,110,111]
Chondroitin sulfate	Cartilage, porcine skin and bovine trachea	N-acetyl-galactosamine and d-glucuronic acid	Dissolving microneedle	Good penetration	[117,118]
Alginate	Brown algae	α-l-guluronic acid and β-d-mannuronic acid	Dissolving microneedle	High mechanical strength when combined with maltose	[136,137]
Xanthan gum	Xanthomonas campestris	β-(1,4)-d-glucopyranose glucan as a backbone with (3,1)-α-linked d-Mann pyranose-(2,1)-β-d-glucuronic acid-(4,1)-β-d-Mann pyranose as side chains	Coated microneedles	Used as viscosity enhancer for coated microneedles	[139,140,152]
Starch	Corn or potato	Glucose	Dissolving microneedle	Owing to its brittleness blended with gelatin	[133,153]
Pullulan	Aureobasidiumpullulans	Maltose	Dissolving microneedle	Exhibited good mechanical properties	[142,154]
Bletilla striata	Bletilla striata	α-mannose, β-mannose, and β-glucose	Dissolving microneedle	Good mechanical strength and sufficient penetrating ability	[146]
Panaxnotoginseng	Panaxnotoginseng	Backbone of→4)-α-d-GalAp-(1→4-β-l-Rhap-1 →4)-β-d-Galp-(1→residues, with a branch of α-l-Araf-1→5)-α-l-Araf-(1→	Dissolving microneedle	Good loading capacity and compatible with hydrophilic and lipophilic molecules, producing sustained and stable drug release	[148]

## 6. The Benefits of Microneedles

### 6.1. Low Cost

Polymeric MN-based natural polysaccharide is less expensive than silicon MNs or metal MNs [155]. Polymers such as chitosan, chitin, hyaluronic acid, sodium alginate and starch are used for the fabrication of microneedles due to their low cost [155]. Furthermore, the current microneedle manufacturing processes need to be enhanced to achieve large-scale production to completely transfer microchip-based microneedles into therapeutic applications. To date, the economic assessments of the technology have not been extensive, but as with any new technology, it is not difficult to expect that the clinical use of MNs will be relatively more costly due to the complicated manufacturing and storage procedures and the long and slow approval process [156].

### 6.2. Flexibility

**Figure 5:**
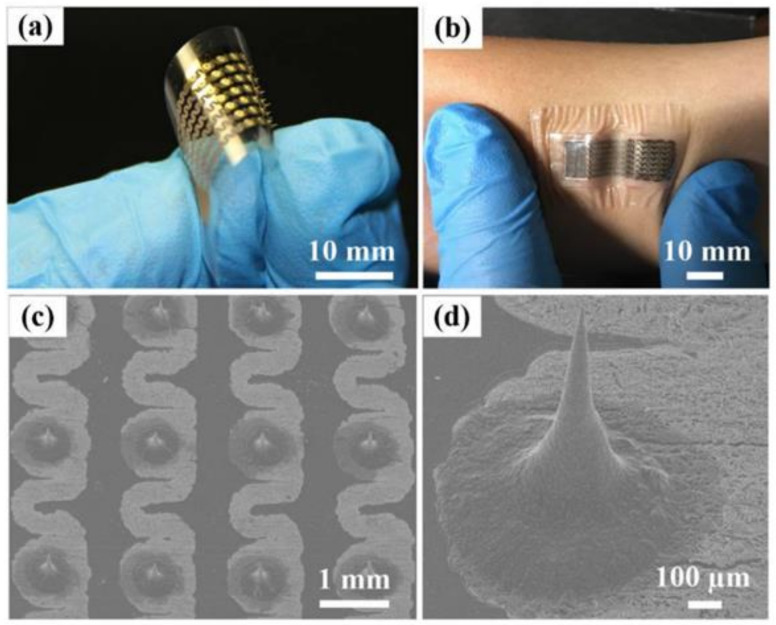
(**a**) Image of the flexible MAE, (**b**) Image of the flexible MAE on the curved skin (**c**) SEM image of the flexible MAE, and (**d**) SEM image of a microneedle (MN). Reprinted with permission from Ref. [159]. Copyright 2019 MDPI.

Since metal and silicon MNs are fragile in nature, they can harm patients. In this case, polymeric MNs are the opposite of silicon MNs. Due to the viscoelastic property of polymers, polymeric MNs have a greater ability to resist shear-induced failure in the skin than silicon or metal MNs (Figure 5).

Ian Woodhouse et al. have developed microneedles that successfully raise oxygen levels by 8–12 ppm when dissolved over a 2-h period, providing a strong bactericidal effect on liquid and biofilm bacterial cultures of gram-positive (Staphylococcus aureus) and gram-negative (Pseudomonas aeruginosa) bacterial strains commonly found in dermal wounds [157]. In addition, the results of ex vivo testing in a porcine wound model have demonstrated the effective insertion of the microneedles into the tissue while offering effective bactericidal properties against both gram-positive and gram-negative bacteria in the complex tissue matrix [158]. In addition, microneedles showed significant levels of cytocompatibility with apoptosis of less than 10% during 6 days of continuous exposure to human dermal fibroblast cells [157]. The flexible microneedle presented may provide a more successful strategy to enhance the effectiveness of topical tissue oxygenation as well as for treating wounds infected with intrinsically antibiotic-resistant biofilms.

### 6.3. Biodegradability, Biocompatibility and Stability

One of the safety aspects of MN systems in clinical use is biocompatibility [36]. To ensure that MN products are suitable for human exposure, different tests are necessary to evaluate their biocompatibility based on contact periods of less than 24, 24 to 30 h, and more than 30 h. For the first two periods, the corresponding tests are cytotoxicity, sensitization, irritation and intracutaneous reactivity tests. Genotoxicity and subacute/sub-chronic systemic toxicity tests are further suggested for the last period of use. The use of biodegradable materials is preferable for microneedles as such materials can be decomposed and be safely disposed of by the body [14]. Consequently, the use of biodegradable polymeric systems for the manufacture of MNs has been investigated in recent years [15]. The main advantage of polymeric microneedle systems is their ability to load drugs into the microneedle matrix for discharge into the skin by biodegradation or dissolution into the skin’s body fluid [160].

The possibility of manufacturing microneedle structures from aqueous polymer blends at room temperature without the need for a warming step could be a significant advantage in retaining the strategy of stability of an incorporated drug, particularly in the case of therapies in which proteins and peptides are implicated [161]. However, the stability of the MN cargo must be evaluated to make sure that fragile and highly degradable therapeutics are protected during the storage process [160]. This is generally performed by studying MNs and their cargo at various temperatures, such as −25, 4, 20, 40, and 60 °C, and then performing analytical measurements. In summary, the protein cargo of MNs has improved storage stability and prolonged shelf life due to the rigid glassy microneedle matrices that constrain molecular mobility and limit the availability of atmospheric oxygen. This period can be prolonged by adding stabilizers, particularly trehalose and sucrose [162]. Careful attention to water is particularly critical when storage conditions are not evacuated, as it can destroy not only the stability of the charged cargo but also the mechanical properties of the MNs. Dissolvable MNs are very sensitive to surrounding moisture; consequently, the storage environment must be dry and cool for prolonged stability and extended storage life [163].

## 7. Conclusions and Future Perspectives

Recently, nanotechnology-based transdermal drug delivery platforms have gained renewed interest. Microneedle (MN) technology shows excellent potential in controlled drug delivery, which has received increasing attention from investigators and clinics, as with the transdermal patch. Cellular delivery, DNA vaccine delivery, skin penetration, local tissue delivery and systemic delivery are enhanced with microneedles and nanoneedles. Thus, microneedles can be manufactured with a variety of modifications to intelligently deliver the drug through the skin, offering a new direction and revolution in the field of transdermal drug delivery systems. This technology thus paves the way for efficient, painless and convenient delivery of medicated drugs and vaccines.

The future of polysaccharides for MNs development depends largely on the development of smart devices for DD, ISF and diagnostics using nanocarriers and nanostructured polymers. Research is actively investigating the creation of MNs that detect changes in pH or temperature or changes in temperature. The advent of nanotechnology may enable the creation of smart diagnostic MNs. As minimally invasive devices, diagnostic MNs could be successful in the clinic. This is an area of enormous potential and is expected to be very popular in the future. It is imperative to emphasize that these MN devices are poised to provide an alternative to conventional oral delivery of pharmaceuticals. Concerning the limitations of the oral route, portability and well-known acceptability to patients must be considered.

In conclusion, the use of elegant structures and remarkable combinations of polysaccharides have allowed the successful delivery of different pharmacological agents using MNs, with a series of applications in various fields of human activity. As such, biopolymer-based MNs are playing a crucial role in modern healthcare. These are on the verge of exciting breakthroughs in the area of DD and may announce an important contribution to drug delivery. In a nutshell, it is anticipated that within the next few years, some MN devices will be validated at the clinical level, taking clues from nature in everyday DD applications.

## Figures and Tables

**Table 1:** Thedecision matrix for the design of suitable microneedles on the following performances presenting as low efficacy (✖), moderate efficacy (▲), and high efficacy (🞅). Reprinted from ref. [21].

	MicroneedleType	Solid Microneedle	Coated Microneedle	Dissolving Microneedle	Hydrogel Microneedle
DecisionParameter					
Drug dose		🞅 High	✖ Low ▲ (If several patches are used)	✖ Low ▲ (If several patches are used)	🞅 High
Onset of action (Pharmacokinetics/ pharmacodynamics)		✖ Slow release by diffusion	🞅 Rapid dissolution	🞅 Dependent on the formulation	✖ Slow release by diffusion
Delivery period		▲ Several hours (agents that keep the pores open longer are additionally needed)	✖ Several minutes	🞅 Several minutes to weeks (depending on the formulation)	▲ Several hours
Delivery efficiency (Expensive drugs require high delivery efficiency)		✖ Some drug remains in the patch or formulation	🞅	🞅	✖ Some drug remains in the patch
Sharp waste generation		🞅	🞅	✖ No sharp waste	▲ Swollen hydrogel microneedle tip
Packaging		▲ Separate packaging for microneedles and formulation	🞅	🞅	🞅
Patch-wearing time		✖ Several hours	🞅 Several minutes	🞅 Several minutes	✖ Several hours

## Data Availability

Data sharing not applicable.
